# Voices of change: experiences of early women urology residents — a single institution qualitative research study

**DOI:** 10.1186/s12909-025-06789-5

**Published:** 2025-02-06

**Authors:** Aurora J. Grutman

**Affiliations:** The Johns Hopkins University School of Medicine, 1600 McElderry Street, Baltimore, MD 21205 USA

**Keywords:** Gender equity, Qualitative research, History of medicine, Medical education

## Abstract

**Objective:**

To understand the experiences of early women urology residents at a single institution, as told in their own words.

**Methods:**

A convenience sample of women residents who trained at the Brady Urological Institute between 1980 and 2022 was chosen. During the investigated period, 15 women trained at the Brady; all 15 were invited to participate. Participants were provided written consent and assured of anonymity. In semi-structured interviews guided by a set of IRB-approved questions, participants discussed personal backgrounds, medical school experiences, and residency experiences. Participants provided advice to future women in urology. Interviews were recorded with Audacity 3.0.5 or Zoom, and audio files were transcribed using NVivo 14.23.0.

**Results:**

A total of 10 interviews were conducted between July 2023 and February 2024. Childhood role models influenced many participants’ initial interest in medicine, although most discovered urology during medical school. Participants valued the quality of training, mentorship opportunities, proximity to family, and program ethos when selecting a residency program. During residency, participants faced gender-specific challenges due to patient resistance and difficulties balancing professional and personal commitments. Despite these challenges, participants expressed optimism about the future of women in urology.

**Conclusions:**

This study provides insights into the professional development of women urology residents. The participants were part of an early cohort of women in urology and expressed excitement for the future of the field. While the study reflects the experiences of women at just one institution, it provides a foundation for more comprehensive research on women’s experiences in urology and in medicine more broadly.

**Clinical trial number:**

Not applicable.

**Supplementary Information:**

The online version contains supplementary material available at 10.1186/s12909-025-06789-5.

## Introduction

In 2023, women comprised 54.6% of all U.S. medical students, 48.2% of all residents, and 37% of all licensed physicians [1–3]. Despite their increasing participation in medicine generally, women currently comprise a minority of resident physicians in several specialties, including orthopedics (20.4%), interventional radiology (22.5%), neurosurgery (23.8%), diagnostic radiology (28.2%), and urology (32.2%) [3].

Although gender parity in urology still lags behind neurology (48.3% female), colorectal surgery (49.0% female), and psychiatry (51.3% female), women’s representation in urology training programs increased 11-fold from 0.9% in 1978 to 23.8% in 2013 [3, 4]. While women’s representation at the trainee level continues to increase– 44.9% of all 2024 urology matches were female– only 11.8% of practicing urologists in 2023 were women [5, 6]. 

While census data quantifies the upward trend of women’s representation in urology, qualitative studies are needed to document female urologists’ experiences during this period of growth. Through semi-structured interviews, this project explores the experiences of women urology residents at a single institution, the Brady Urological Institute at Johns Hopkins, from 1980 to 2022. Methodologically, this study borrows from oral history traditions to capture stories of the “past seen through the eyes of the present with an eye on the future,” as described by oral historian Sylvia Lubow [7]. The study is suggestive and not representative. By documenting and preserving these women’s stories, in their own words, this study may inform future initiatives to enhance gender equity in urology and contribute to ongoing conversations about women’s growing representation in medicine.

## Materials and methods

This study was approved by the Johns Hopkins Institutional Review Board (IRB) in June 2023. The questions were developed based on validated interview questions from other qualitative studies of women in medicine [8–11]. Questions were approved by the IRB prior to interviews (Supplemental). The semi-structured interview methodology allowed for flexibility in the response format, such that additional questions could be asked related to participant responses [12, 13]. 

**Fig. 1:**
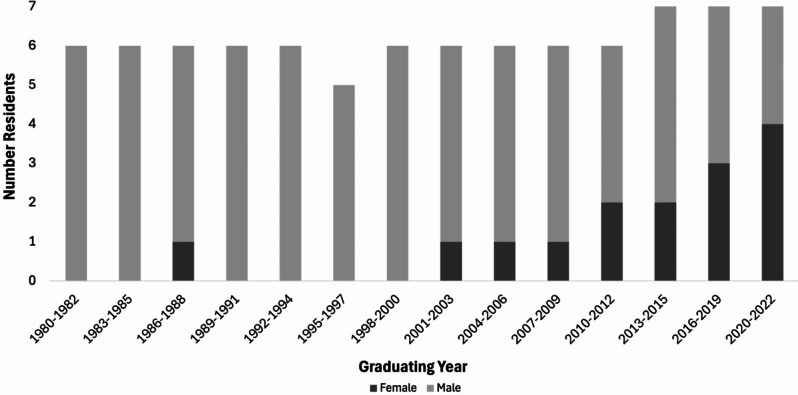
Timeline of Women’s Participation in the Brady Urological Institute Residency Program (1980–2022)

A convenience sample of women residents who trained at the Brady Urological Institute was selected because institutional records allowed easy identification of potential interviewees and contact information could be obtained. Given IRB concerns about potential disturbances to the workplace environment caused by interviews with current residents, 1980 and 2022, which correspond to the arrival of the first woman resident and members of a recent graduating class, respectively, were selected as temporal boundaries. During this period, 92 total residents trained at the Brady; 15 (16%) were women (Fig. 1). All 15 were invited to participate; 2 did not respond to inquiries; 3 declined; 10 agreed.

Before each interview, participants provided written informed consent and were assured of anonymity. Interviewees were not compensated for their participation. Interviews were conducted by a single researcher (AG) who had no prior relationship with the participants. The selection of a single interviewer reduced inter-interviewer bias by ensuring a similar questioning style, tone, and interpretation of responses. Time constraints limited the number of questions that could be asked in each interview. However, all participants were asked questions from four general categories: personal background, medical school experiences, residency experiences, and career reflections. Interviews were recorded with Audacity 3.0.5 or Zoom, and audio files were transcribed using NVivo 14.23.0 and manually checked for errors. Transcripts were thoroughly evaluated for commonly discussed experiences among participants. Representative quotes were selected from responses to each question category, with efforts made to ensure the speaker could not be identified from the selected quote.

Due to imprecision in the literature, the terms “gender” (women/men) and “sex” (female/male) are often used interchangeably [10]. In this study, both terms are used. To be sure, the author recognizes that sex, gender, and gender identity are distinct categories and supports continued efforts to standardize language and improve inclusivity.

## Results

Of the 15 women residents who trained at the Brady between 1980 and 2022, 10 (66.7%) participated in semi-structured interviews conducted in person or via Zoom between July 2023 and February 2024. The interviews ranged from 47 to 77 min (mean = 65 min). Demographic details are not shared to preserve participant anonymity. Representative quotes from each of the four general categories are presented in Tables 1, 2, 3 and 4. Given the weighted priority of questions related to residency experiences, quotes pertaining to gender-specific challenges and cultivation of work-life balance are presented in Tables 3 and 4. These tables present a selection of quotations that the researcher subjectively evaluated as representative of the views of several interviewees.

**Table 1:**
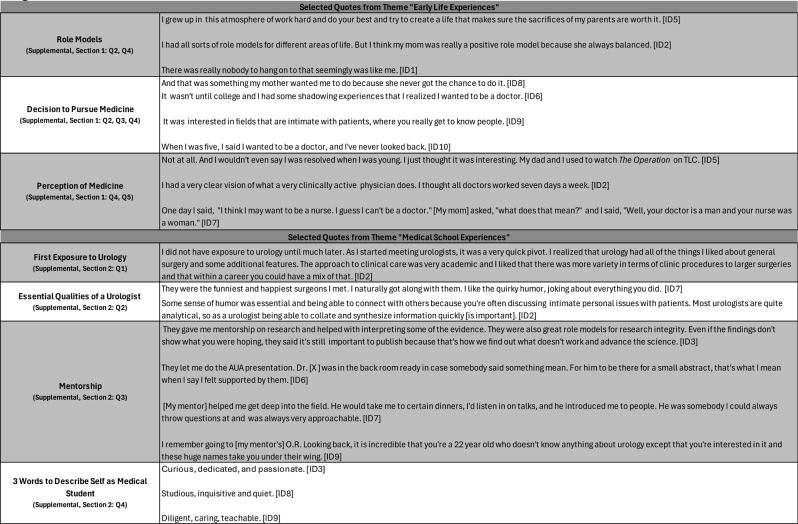
Selected quotes from themes “Early life Experiences” and “Medical School Experiences”

### Personal background

Early childhood experiences contextualized participants’ interests in medicine (Table 1). Many participants described formative role models who helped shape their path to becoming a doctor, and three participants explicitly mentioned parents or other family members who set high expectations for career goals. Most participants did not develop an interest in medicine until college (4/10), but identified an early passion for math, science, or connecting with others. For the few interviewees who wanted to be a doctor since childhood, early exposure to medicine was cited as a shaping force. Although most participants did not have a family member in medicine, six participants shared that a mother or grandmother either was a doctor or had wanted to become a doctor but did not. The majority of participants (8/10) self-evaluated that, prior to medical school, they did not understand what a medical career would hold but were eager to learn more.

### Medical school experiences

Nearly all participants (9/10) discovered urology during medical school (Table 1). The combination of clinical and surgical duties was appealing to many participants. Participants also appreciated what they identified as a urologist personality type. During medical school, nine interviewees had close relationships with mentors. For the majority of participants, these mentors were in the field of urology. Mentors guided research endeavors and supported students in and outside of the operating room. Although most of the participants were early in their careers, they were a passionate, driven, and curious group excited about a future in urology.

### Choosing a residency program and early residency experiences

Participants identified factors important in selecting a residency program (Table 2). Quality of training (2/10), geography and/or proximity to family (4/10), likelihood of receiving strong mentorship (2/10), camaraderie and communal cohesion (3/10), and foundational training opportunities for future success (2/10) were mentioned as top priorities. The transition to becoming a Brady resident was overwhelming for many. However, participants adapted in a short time. Mentors took the form of senior residents and faculty members who invested in and supported the participants.

**Table 2:**
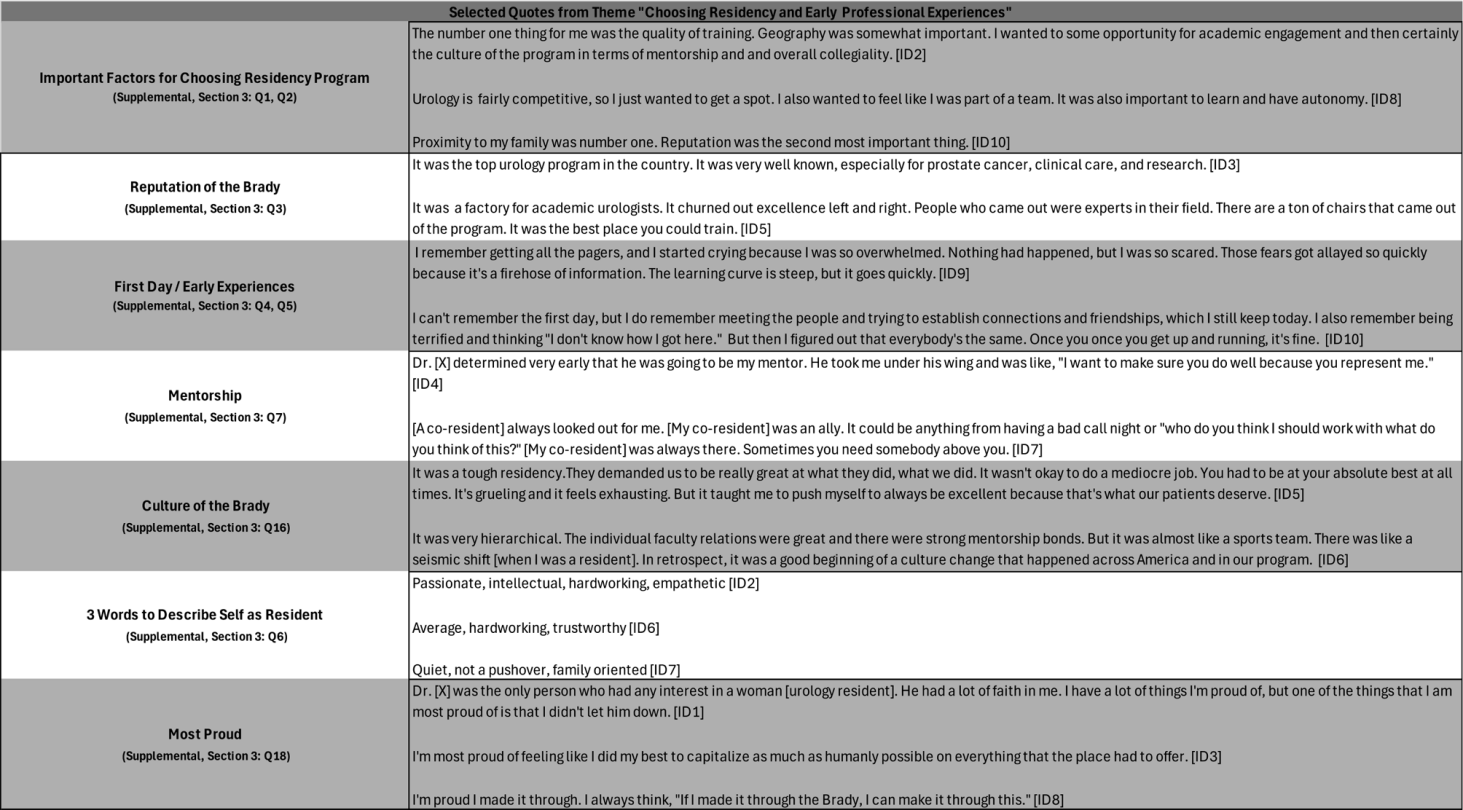
Selected quotes from themes “Choosing residency and early professional Experiences”

Participants reported that the Brady culture was defined by its commitment to excellence and high expectations. Faculty members who themselves had trained at Brady had especially high expectations for what each resident should be capable of surgically, clinically, and academically. Some participants (4/10) discussed programmatic shifts at Brady; these shifts in some way involved restructuring of the medical hierarchy and corresponding expectations for residents and faculty.

Participants described themselves as committed, meticulous, and empathetic during residency. Of their accomplishments during residency, participants were most proud of developing surgical capacities (3/10), cultivating work-life balance (2/10), taking care of patients (1/10), completing the program (2/10), and improving the communal ethos (1/10).

### Gender-related experiences during Residency

Participants described how gender shaped interactions with faculty, peers, and patients (Table 3). One participant observed that an error by any resident, regardless of gender, would earn rebuke from the faculty. Another participant did not feel she was treated differently from male residents but wondered whether her “aggressive personality” may have shaped interactions with faculty members. Other participants identified that occasionally women residents were compared explicitly to other women residents, and that faculty members sometimes made gendered comments. Nevertheless, participants did not feel they were treated differently overall because of their gender. Participants described interactions with peers as largely collaborative. Instances of conflict was evaluated as an inevitable by-product of both constant work and differing viewpoints.

**Table 3:**
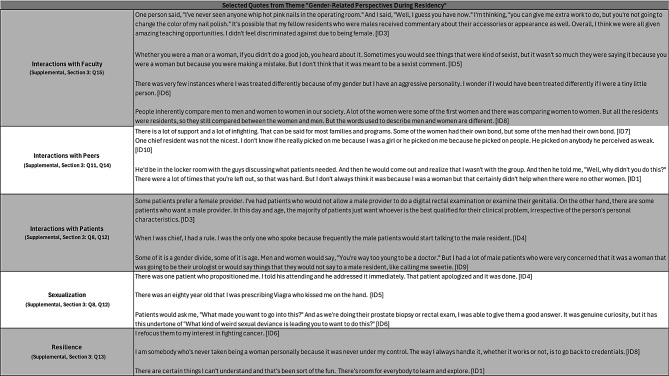
Selected quotes from theme “Gender-Related perspectives during Residency”

Most participants (7/10) identified gender-related resistance from patients who often preferred a male provider. Some male patients spoke differently to women residents, using terms like “honey” or “sweetie,” or speaking exclusively to male residents in the presence of both male and female residents. Two participants discussed how patient age may have contributed to patient resistance to a female urologist (with older patients being more likely to resist a younger provider). Some participants (4/10) described interactions with male patients they viewed as inappropriate. Interviewees leveraged their professional capabilities and used various coping strategies to contextualize gender-related resistance from patients.

### Cultivation of work-life balance during Residency

During residency, all participants found it difficult to balance clinical responsibilities with personal commitments, interests, and relationships (Table 4). Most participants (8/10) stated that support from fellow residents who understood the demands associated with surgical training helped them “not fall apart.”

**Table 4:**
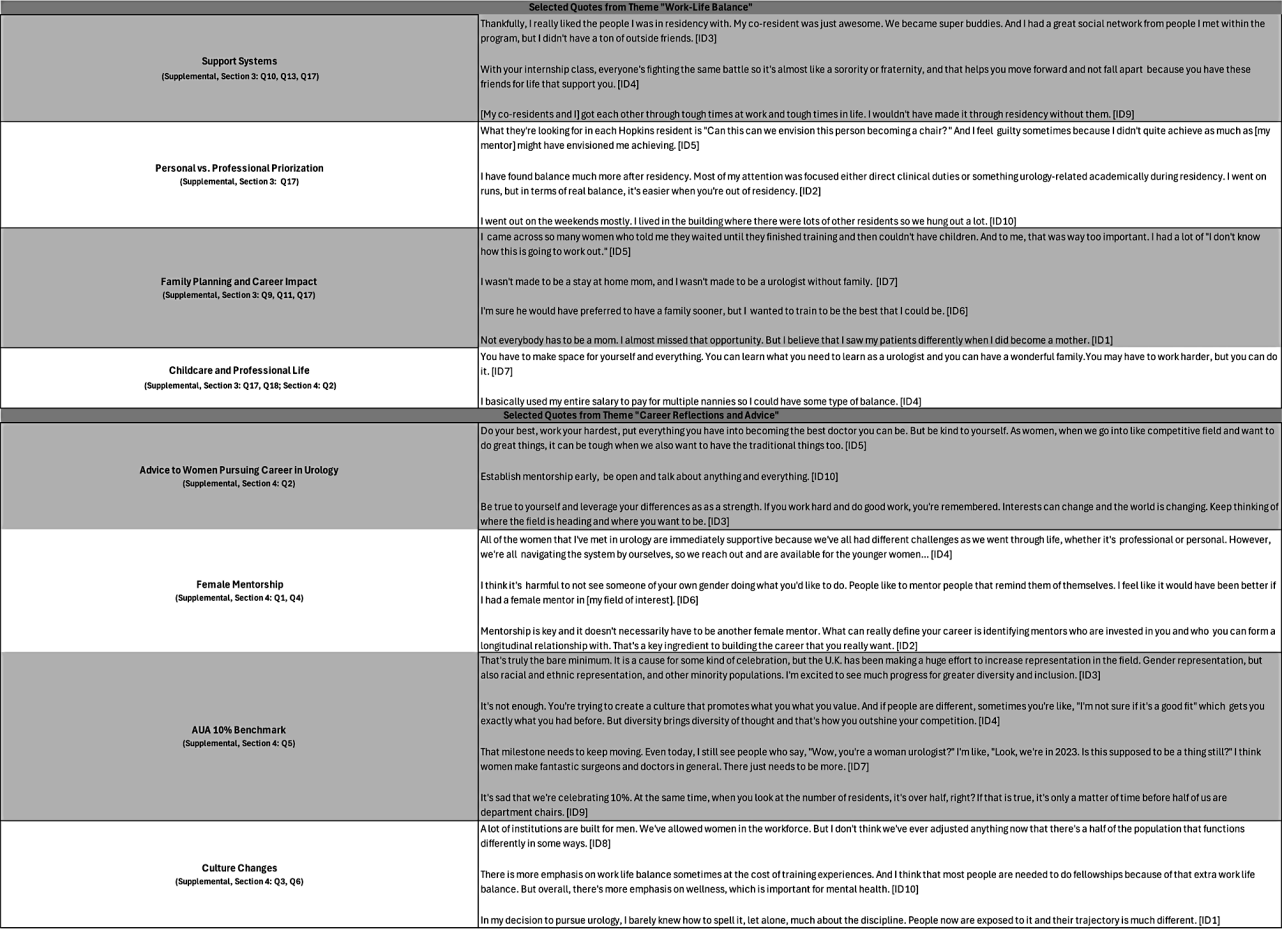
Selected quotes from themes “Work-Life Balance” and “Career reflections and Advice”

Some found it challenging to shift their focus away from urology-related matters. For one participant, finding work-life balance during residency was her greatest accomplishment. For others, balance took the form of exercising or socializing on the weekend. Some participants desired a family, which required recalibrating professional and personal commitments.

Regardless of any one participant’s interest in starting a family during residency, many reflected on the various challenges posed by pregnancy during surgical training. However, motherhood provided some participants with a new perspective that fostered greater connection with patients.

### Career reflections and advice

Participants reflected on their career development and provided advice to future generations of women in urology (Table 4). Participants encouraged women to pursue urology, emphasizing the importance of fully applying oneself, connecting with mentors, and reflecting on goals.

While many praised their male mentors, three participants underscored the value of female mentorship. When reflecting on women’s growing representation in urology, participants remarked that any celebration of women’s comprising more than 10% of the urology workforce for the first time in 2020 was “sad,” “not enough,” and “pathetic.”

Participants highlighted various cultural changes in the field of urology, in addition to women’s increased representation, such as greater emphasis on work-life balance. Two participants cautioned that work-life balance should not come at a cost to quality patient care. Overall, participants were optimistic about the future for women in urology.

## Discussion

This study captured influential moments in the professional development of women urology residents training at a single institution between 1980 and 2022. Semi-structured interviews helped identify shared experiences among these women, such as the importance of mentorship and representation, difficulties with work-life balance, and the role of gender in interactions with colleagues and patients. Frequently discussed experiences, without claiming to be representative, nevertheless provide a catalyst for further conversation about the evolution of women’s inclusion in urology and the need for continued growth.

For many participants, the transition to residency was challenging. New clinical responsibilities and high faculty expectations left many participants anxious, nervous, and doubtful of whether they were qualified–common feelings among first-year surgical residents generally [14]. Feelings of self-doubt and imposter syndrome, which are widely reported among medical trainees and women in surgery, in particular, were pervasive in the early days of residency [15, 16]. During the transition, mentorship emerged as a cornerstone of support. At the Brady, faculty members recognized resident talent and invested early, encouraging growth in and outside of the operating room. Participants confided in their mentors, seeking advice about career development, difficult patient cases, and research. However, mentorship often came with an unstated expectation for “greatness,” leaving many trainees doubtful of their ability to achieve success, as defined by predominantly male mentors. Particularly for women training at a time when there were few other women in urology, these expectations carried substantial weight: “I knew I was given an opportunity that others didn’t have, and I knew if I did not shine, then others wouldn’t have it in the future.” The pressure and privilege of mentorship was something female trainees did not take lightly.

Avenues for mentorship at the Brady were often informal, prompted by recognition of talent and consistent cultivation of skills and interests. Indeed, formal mentorship programs are not universal in urology. In the United States, approximately 58% of urology residencies have a formalized mentorship program; only 5% have a specific mentorship course for faculty [17, 18]. Research has shown formal mentorship programs are beneficial for productivity, overall purpose, and equity. Considering the trajectory of women’s involvement in urology– in 1978, less than 1% of urology residents were female– mentorship organizations such as the Society of Women in Urology have been instrumental in connecting women urologists, providing opportunities for exchange about professional goals, and concerns about balancing work and personal life [4, 19]. As the field continues to expand to include more women, the need for explicit mentorship networks becomes clear. For participants, male mentors proved to be some of the participants’ fiercest advocates in the face of skepticism. However, many interviewees wished they had a female role model who could provide advice specific to women, such as becoming a mother, which remained a persistent challenge for participants.

For some participants, demands of professional excellence, which required a singular focus on training, directly conflicted with a desire to have children. Participants reflected concerns about the feasibility of pregnancy, a subjective assessment confirmed by studies showing women in urology have more fertility complications than peer physicians and American women in general [20]. Female urologists are less likely to have children, and if they do, they work more hours while pregnant and take shorter maternal leaves than other female physicians [20]. While many institutions, including the Brady, have formal parental leave policies, there is no widespread agreement on adapting training programs to effectively and equitably shift professional commitments when a urologist becomes pregnant [21]. Without established programs and policies, the feasibility of pregnancy during medical residency generally and urology training in particular remains uncertain.

The study also revealed how gender biases permeated patient interactions. Many participants reported skepticism from male patients, who preferred male providers entirely or addressed female residents with gendered terms such as “honey” or “sweetie.” On several occasions in their interactions with male patients, participants faced implicit or explicit sexualization. This is a common experience in urology, with one survey indicating 78% of women urology residents in the U.S. experienced harassment from male patients [22]. While participants did not feel they were treated differently from their male counterparts by faculty or peers, this is not the experience of women surgical residents generally [23]. Despite increasing participation in urology, women trainees continue to face high rates of bias and harassment, highlighting the need for education campaigns about implicit bias and institutional mechanisms to address inappropriate behavior [22–25]. 

The field of urology has undoubtedly changed since the Brady accepted its first woman resident in 1980. Interviewees believed women are more accepted in urology today, but resistance and skepticism remain, especially from male patients. Nevertheless, interviewees were optimistic about a future when women eventually will become well represented in leadership positions, such as program directors and department chairs, which in turn will attract more women to the field.

These findings are limited by the number of interviews conducted and not all women who trained at the Brady between 1980 and 2022 were interviewed. Moreover, findings describe the experience of women trainees who trained at a single institution in the United States. This study’s semi-structured interviews did not seek to quantify attitudes or experiences. During interviews, participants were not obligated to answer any question, and not every question was asked, given time limitations. Additionally, the retrospective approach introduces recall bias, and the sensitivity of specific questions may have led to reporting bias. Lastly, this article centers around women’s experience of gender, without delving into other intersecting identities such as race, sexual orientation, and socioeconomic status, which also shape experiences in urology. The study did not also seek to compare women’s experiences explicitly to men’s.

Although conducted at a single institution, this study captured themes and experiences likely shared by women urologists training at other time periods and at different institutions. Mentorship, work-life balance, and gender dynamics appear to remain central to the experience of women in the field, and future studies should adopt intersectional lenses to explore how varying aspects of identity overlap and intersect to influence residents’ training experience. Only with greater understanding of these lived experiences can strategies for increasing diversity in the field be developed and actualized. Such efforts are critical for cultivating the next generation of urologists which includes more women.

## Conclusion

Women who trained at Brady Urological Institute from 1980 to 2022 helped expand gender representation in urology. Participants discussed the positive influence of mentors on their careers, shared difficulties related to work-life balance, and described resistance from some patients. Because this study presents a single-institution perspective, it sets the stage for further research to more comprehensively study women’s experiences in urology. The history of women’s participation at the Brady is part of a larger story of women’s inclusion and success in the field of urology. And that story is just beginning.

## Electronic supplementary material

Below is the link to the electronic supplementary material.

Supplementary Material 1

## Data Availability

Data provided within this manuscript was obtained from questions provided in the supplementary information. The data is securely deposited with the researcher and may not be accessed to ensure privacy to participants.

## Abbreviations

IRB: Institutional Review Board
