# Supplementary material for: Voices of change: experiences of early women urology residents — a single institution qualitative research study
Source: BMC Med Educ. 2025 Feb 6;25:194. doi: 10.1186/s12909-025-06789-5 (PMC11803943; doi:10.1186/s12909-025-06789-5)
Supplement: Supplementary file 1 — Supplementary Material 1 [file 12909_2025_6789_MOESM1_ESM.docx]

**Section 1: Background and Upbringing**

1. When and where were you born?
2. Could you tell me a little bit about your family? What did your parents do?
3. Did you have any aspirations (the classical “what do you want to be?”) as a child?
4. Did you have any role models growing up?
5. When you were younger, what did you think a doctor did?

**Section 2: Medical School Experiences**

1. When did you first learn about urology? What drew you to the discipline?
2. Which qualities did you think were essential to being a urologist?
3. Did you have any mentors during medical school? Did they influence your decision to pursue urology?
4. Provide three words to describe yourself as a medical student.

**Section 3: Residency Experiences**

1. What factors were important to you when choosing a residency program?
2. What were you particularly excited about when thinking about residency? Concerns?
3. What was the reputation of the Brady? What had you heard from other medical students? Residents?
4. Do you have any recollection of your first day of residency? Could you share any memories you have?
5. What was a day in the life as a resident at the Brady like?
6. Provide three words to describe yourself as a resident.
7. Who were your earliest mentors? What did you learn from them?
8. Do any specific interactions with patients stick out to you? If so, could you describe them?
9. What did you find easy during residency? What was more difficult?
10. What were your peers like? What was the nature of collaboration like? What it competitive? How was it among men? Between men and women? Among women?
11. Were there any instances of resistance because you were underrepresented in the field?
12. Did you notice any differences in your interactions with male and female patients?
13. In moments where you experienced resistance, how did you stay motivated? What did/does resiliency look like for you?
14. Did you notice any differences between how you and your male peers approached patient cases or surgical procedures?
15. How did members of the faculty treat residents?
16. What was the culture like at the Brady?
17. How did you find balance between your personal life outside of medicine and professional life during residency?
18. Looking back on your experience during residency, what are you most proud of?

**Section 4: Career Reflections and Advice**

1. When you decided to pursue a career in urology, did you have anyone you could go to for advice? How did you navigate the process?
2. What advice would you give to women pursuing a career in urology?
3. What expectations do you think people have for women in surgery? Women in urology? Have these expectations changed over time?
4. What role, if any, has female mentorship or peer support played in your professional life?
5. In 2021, the AUA celebrated women comprising more than 10% of the urology workforce. What feelings did you have about that milestone?
6. Has the overall culture of urology changed since you began residency?
